# Squalene Supplementation as a Novel to Increase PUFA Content in Fish Tissues

**DOI:** 10.3390/ani13162600

**Published:** 2023-08-11

**Authors:** Piotr Niewiadomski, Piotr Gomułka, Małgorzata Woźniak, Mariusz Szmyt, Elżbieta Ziomek, Helena Bober, Mirosław Szczepkowski, Katarzyna Palińska-Żarska, Sławomir Krejszeff, Daniel Żarski

**Affiliations:** 1 Department of Ichthyology and Aquaculture, University of Warmia and Mazury, 10-718 Olsztyn, Poland; 2 Department of Tourism, Recreation and Ecology, University of Warmia and Mazury, 10-718 Olsztyn, Poland; mawoz@uwm.edu.pl; 3 Department of Sturgeon Fish Breeding in Pieczarki, The Stanislaw Sakowicz Inland Fisheries Institute, 11-610 Pozezdrze, Poland; 4 Department of Ichthyology, Hydrobiology and Aquatic Ecology, The Stanislaw Sakowicz Inland Fisheries Institute, 10-719 Olsztyn, Poland; 5 Department of Aquaculture, The Stanislaw Sakowicz Inland Fisheries Institute, 10-719 Olsztyn, Poland; 6 Department of Gametes and Embryo Biology, Institute of Animal Reproduction and Food Research, Polish Academy of Sciences, 10-748 Olsztyn, Poland

**Keywords:** Eurasian perch, Siberian sturgeon, rainbow trout, antioxidant, health status, fatty acids profile, squalene

## Abstract

**Simple Summary:**

The study aimed to assess the effect of squalene supplied in feed on the growth performance, health status, and fatty acid profiles of the muscle and liver of Siberian sturgeon, rainbow trout, and Eurasian perch. To achieve our aim, we fed fish with three feeds containing different levels of squalene (0%, 0,5%, 1%). Then, analyses such as hematological and biochemical assays, liver histology, and fatty acid content of muscle and liver were performed. The results that we obtained indicate changes in the values of hematological, and biochemical indicators between groups. Squalene addition influences the nucleocytoplasmic index values in all fish offered feed containing 1% squalene. The PUFA and docosahexaenoic acid increased with both groups where squalene was added. Exogenous squalene increases the content of PUFAs in the liver and muscles of the examined species.

**Abstract:**

Squalene is an antioxidant that plays an essential role in fat metabolism. The study aimed to assess the effect of squalene supplied in feed on the growth performance, health status, and fatty acid profiles of muscle and liver of Siberian sturgeon, rainbow trout, and Eurasian perch. The experimental feeds containing 0%, 0.5%, and 1.0% squalene were prepared for each fish species. Hematological and biochemical indices, liver histology, and fatty acid profiling of muscle and liver were analyzed. Squalene supplementation was safe for fish, and no negative influence on growth status was observed. However, changes in the values of hematological and biochemical indicators were found, including the level of triglycerides in the blood of rainbow trout, and cholesterol in the blood of Eurasian perch. The addition of squalene influences the nucleocytoplasmic index values in all fish offered feed containing 1% squalene. The retention of squalene in the liver and muscle of experimental Siberian sturgeon was observed in both 0.5% and 1.0% squalene levels of feed. The PUFA and docosahexaenoic acid increase was observed in all fish in groups with squalene addition. Dietary squalene increases the content of PUFAs in tissues of the examined species.

## 1. Introduction

The decreased availability and growing prices of fish meal (FM) and fish oil (FO) have become challenging for the aquaculture industry [1] and have prompted the search for alternative sources of proteins and fat [2]. The replacement of FM with alternative plant protein sources remains a major area of research and much has been accomplished in reducing the level of fish meal in the nutrition of all fish species [3]. The supply of FO depends on the amount of FM obtained. FO is, in tsurn, the main source of n-3 polyunsaturated fatty acids (PUFA); alternative solutions are still limited [4]. It is now well established that fish, similarly to all vertebrates, cannot synthesize de novo PUFA and consequently, that essential fatty acids (EFA) must be provided to them in their diet [5]. Tocher [6] stated that because of the need for a supply of n-3 PUFA, the continued growth of aquaculture is dependent upon the development of more sustainable feeds with alternative sources of these fatty acids. Supplementation with vegetable oils in fish feed lowered the content of docosahexaenoic acids (DHA) in fillets compared to feed with FO [7]. That is why it is crucial to look for new solutions limiting the consumption of FO in aquaculture and other species of fish, including maintaining the quality of fish meat at this DHA level.

According to Tocher [4] it is largely related to the easy oxidation of these fatty acids in biochemical reactions taking place in the body cells. However, is hypothesized that protecting PUFA against oxidation in fish bodies may be a novel method of increasing PUFA content in fish tissues. Many different antioxidants were studied during the last decades. The most promising one to be used in aquaculture is squalene (SQ) as it naturally occurs in fish.

The SQ is a hydrocarbon chain formed by six isoprene units assembled in the form of triterpene which gives SQ the lipid character. The six carbon double bonds (C=C) allow the molecule to be one of the most unsaturated lipids and are responsible for its considerable reactivity [8]. The exogenous SQ probably contributes to the oxidative stability of the body [9]. It is known that squalene is a potent antioxidant [10]. Its antioxidative action is based on catching free radicals, especially reactive oxygen species (ROS). ROS reacts with SQ double bonds (C=C) and it stabilizes by forming a squalene hydroperoxide (HOO-SQ). This reaction runs in accompaniment with creating many SQ isomers [10]. Due to squalene’s strong antioxidant properties, the determination of the optimal level of it in the diet of each species is very important. It will allow squalene to play an antioxidative role and not deteriorate the specimen’s health status at the same time.

SQ is not commonly used in commercial animal feeds, but some experimental studies deal with squalene as an animal diet supplement. Several of them focused on the health-promoting properties of SQ supplementation in animal models. Those studies investigate the effects of SQ supplementation on various organ dysfunctions, ailments, and biochemical indicators in the animal models, namely rats, hamsters, chickens, or pigs [11,12,13,14]. Some of them also take into consideration the growth performance, health status, and meat quality of experimental specimens. One of them is a study performed by Gao et al. [15] which concluded that the SQ effectively increases the growth of piglets, protects good gut microbiota, and improves the antioxidant capacity of the blood. The other group of scientists performed a study on broiler chickens and they also reported a positive influence of SQ on growth, antioxidative status, and meat quality [16]. Moreover, another type of study considering reproduction improvement was performed on meat-type male chickens, which were supplemented with SQ to obtain a better reproductive performance. In this experiment, satisfying results were obtained after SQ supplementation as well [17].

All of the mentioned studies carried out on various species of animals show health, growth-promoting, and antioxidative features of squalene. However, there is no information on whether the dietary squalene may play the role of an antioxidant in fish, and consequently increase the presence of PUFAs and DHA in different tissues. Some of the research by Dessì [18] allows us to conclude that SQ supplementation may protect PUFA and DHA against oxidation in fish in vivo and thus the share of PUFA in supplemented fish should be higher in comparison to control ones.

This study aimed to investigate the effect of dietary squalene on growth performance, health status, and fatty acid profiles in the muscle and liver of Siberian sturgeon, rainbow trout, and Eurasian perch.

## 2. Materials and Methods

### 2.1. Ethics Statement

Studies on live animals were carried out in strict accordance with the recommendations of the National Ethics Commission. According to Polish law and an EU directive (no 2010/63/EU), the experiments conducted in this study did not require an ethical approval certificate.

### 2.2. Fish

The experimental fish were juvenile Siberian sturgeon (E1) obtained at the fish farm of Inland Fisheries Institute in Olsztyn in Pieczarki (Pozedrze, Poland) (21°47′50.280″ E, 54°6′49.392″ N), rainbow trout (E2) from the fish farm Czarci Jar (Czarci Jar, Poland) (20°9′49.458″ E, 53°34′1.168” N) and Eurasian perch (E3) at the fish farm of the Les Perches Lucas (Hampont, France) (6°34′56.144” E, 48°50′6.543″ N). Siberian sturgeon with an initial mean body weight of 66.7 ± 12.7 g and mean total length of 27.8 ± 1.6 cm, rainbow trout of 26.6 ± 5.7; 13.3 ± 1.0 cm, and Eurasian Perch 23.75 ± 4.50 g; 11.5 ± 0.5 were used in experiments.

### 2.3. Experimental System

In this study three separate experiments were performed on the following species: Siberian sturgeon (E1), rainbow trout (E2), and Eurasian perch (E3). Experimental fish were transported to the Centre of Aquaculture and Ecological Engineering in Olsztyn (20°27′43.203” E, 53°45′12.382″ N). Before the beginning of experiments, fish were caught and immediately anesthetized with MS-222 (Finquel, Argent Laboratories, Redmond, WA, USA) for Siberian sturgeon 125 mg dm^−3^ [19], for rainbow trout 150 mg dm^−3^ [20], and 150 mg dm^−3^ Eurasian perch [21]. For each experiment, we used 180 individuals per species. Three experiments were performed on the same recirculation system (RAS) in different environmental conditions which contained different diets made directly to meet the requirements of each species. Before the start of each experiment, fish were randomly distributed into 9 tanks (3 groups in triplicates; n = 20). Then fish were acclimated for 14 days to the experimental conditions. Each tank volume was 300 dm^3^. Fish were exposed to an artificial light regime of approximately (E1) 12:12 LD (light day): DD (dark day); (E2) 8:16 LD:DD and (E3) 24:0 LD:DD. Water quality parameters were measured every day (8.00 a.m.) and the mean values were as follows: dissolved oxygen > 7.87 mg dm^−3^, temperature: (E1) 22.06 ± 1.10 °C; (E2) 16.14 ± 0.34 °C; (E3) 23.32 ± 0.65 °C, pH 7.50–7.92, total ammonia < 0.005 mg dm^−3^, nitrate < 0.20 mg dm^−3^, nitrite < 0.002 mg dm^−3^ and phosphates < 0.005 mg dm^−3^.

Daily weight gain was calculated based on the increase in biomass of fish fed the control diet during their acclimation period (2 weeks). Feeds were offered at a ratio of 1.3% (E2) and 2.0% (E1 and E3) of fish biomass. Fish were fed by an automatic dispenser. Each of the nutritional experiments lasted for 8 weeks [22].

### 2.4. Diet Formulation & Preparation

Fish were fed with 3 experimental diets S0, S0.5, and S1.0 [12] containing 0%, 0.5%, and 1.0% of squalene (SIGMA-ALDRICH, Germany), respectively. The diets were formulated to meet Hung’s [23]—E1; [22]—E2 and [24]—E3 requirements for all indispensable amino acids and other nutrients. The chemical composition of the experimental diets is shown in Table 1. The fatty acids profiles of the components and experimental diets are presented in Table 2. All diets were isoenergetic and isonitrogenous.

All dry ingredients were ground in a hammer mill, sieved through a 0.8 mm screen, mixed, preconditioned, and extruded through a single screw extruder (METALCHEM, Poland) with a 2.0 mm die. The extrusion processing parameters were controlled during feed production: temperature in the conditioner outlet, 95 °C; temperature in the second segment, 110 °C; temperature in the endplate, 120 °C; pressure at the endplate, 15 bar; a revolution of the screw, 85 rpm; a revolution of a cutter, 70 rpm. The content of total oil (TO) and squalene (SQ) was added to the “raw feed” after the extrusion process. All the “raw feed” was prepared at the beginning of the trial. The feed was stored in a refrigerator inaccessible to UV at 4 °C.

### 2.5. Chemical Analysis

Before chemical analysis, 15 fish from each group (S0, S0.5, and S1.0) were caught randomly, and immediately euthanized by overexposure to the MS-222 solution (300 mg dm^−3^). The contents of the basic chemical components of feed, muscle, and liver (dry matter, crude protein, fat, and ash) were determined by standard methods [25]. Gross energy was calculated as described by NRC [22]. Dry matter was determined by drying in an oven at 105 °C for 24 h. Total protein was determined by Kjeldahl’s method and crude fat by Soxhlet’s method. Crude ash content was determined gravimetrically following a loss of mass after the combustion of the sample in a muffle furnace at 550 °C for 12 h. Nitrogen-free extract (NFE) was calculated as follows: NFE (%) = 100 − (moisture% + protein% + lipid% + ash% + fiber%) [26].

Fish were filleted, skinned, and pooled (3 samples per tank) for further analysis. The fatty acids (FA) composition of fish muscle and liver, experimental feeds, and components was determined by gas chromatography. Quantitative and qualitative analyses of FA were performed on cold-extracted lipids according to the method described by Folch et al. [27]. The fatty acids were methylated by adding a mixture of chloroform, nonaqueous methanol, and sulfuric acid (100:100:1) [28] to 50 mg of a fat sample and heated in a boiling water bath [29]. The mixed assay method was applied for squalene determination. The mixture was extracted with hexane and an aqueous KOH solution [30]. Then, the FA profile was analyzed by using an HP 6890 N gas chromatograph (HP, Germany) equipped with a split ejector (split ratio 1:50) and a flame ionization detector. The quantity of FA was evaluated by comparing its peak area and that of the internal standard. A capillary gas chromatograph was fitted with a fused silica capillary column (HP-INNOWAX, Agilent Technologies, USA; 30 m length, 0.32 mm internal diameter, and 0.25 μm film thickness) with helium (FA) and nitrogen (SQ) as a carrier gas. The recommendations for the chromatographic analysis were applied [31] for FA and [32] for squalene.

### 2.6. Growth Measurements

Growth performance indices were determined as follows:

Specific growth rate (SGR) = [(ln final weight − ln initial weight)/time (days) × 100];

Feed conversion ratio (FCR) = feed intake/wet weight gain;

Hepatosomatic index (HSI) = liver weight/body weight × 100;

Viscerosomatic index (VSI) = (digest tract weight/final body weight) × 100;

Protein efficiency ratio (PER) = live weight gain (g)/crude protein fed (g)

### 2.7. Hematology

Blood samples (n = 15 per group) were collected with a syringe covered with heparin lithium salt from caudal vessels. The hemoglobin concentration in fish blood was determined by Drabkin’s method using an automatic luminometer (TECAN Infinity M200 Pro; Männedorf, Switzerland). The following hematological indices were determined according to Svobodova et al. [33]: red blood cell count (RBC), packed cell volume (PCV), hemoglobin concentration (Hb), mean cell volume (MCV), mean hemoglobin content (MCH) and mean cell hemoglobin concentration (MCHC).

### 2.8. Biochemistry Indices

Blood samples (n = 15 per group) were centrifuged with StatSpin centrifuge for 30 s at 15,800 rpm. Plasma samples were analyzed with a Catalyst Dx Chemistry Analyzer (Idexx Lab; Westbrook, ME, USA) using dedicated test slides (custom panels). The following biochemical measurements were performed: glucose (GLU), triglycerides (TAG), cholesterol (CHOL), total protein (TP), albumins (ALB), globulins (GLOB), and aspartate aminotransferase (AST). Each plasma sample was thawed only once at room temperature, and all the above measurements were performed at once to eliminate multiple freeze/thaw cycles.

### 2.9. Hepatocyte Measurements

The collected liver samples (n = 15 per group) were fixed in Bouin’s fixative solution, dehydrated in ethanol, cleared in xylene, embedded in paraffin blocks, and then sliced into 4–5 μm sections with a RM 2155 rotational microtome (LEICA Microsystems, Wetzlar, Germany). Cross-sections of tissues were stained with hematoxylin and eosin (H&E) [34]. For additional cytological characterization of the liver, the diameters of 50 hepatocytes and their nuclei from each specimen were measured. The number of hepatocytes was established in a field with a surface area of 2500 μm^2^ (50 × 50 μm). Twenty such fields were measured for each individual. Measurements of hepatocyte cell diameter (HCD) and hepatocyte nuclear diameter (HND) were performed with a LEICA DM 2500 light microscope (LEICA, St Gallen, Switzerland) and AxioVision 4.8 computer analysis software (Zeiss, Oberkochen, Germany). The nucleocytoplasmic index (NCPI) was calculated as follows: NCPI = HND HCD-1.

### 2.10. Statistical Analysis

The normality of the data distribution was tested by using the Shapiro-Wilk test and the homogeneity variances by using Levene’s test. When the above assumptions were met, the statistical significance of differences among the means was analyzed using ANOVA and Tukey’s post-hoc test (TT). For the others, Kruskal-Wallis ANOVA (KWT) and the Mann-Whitney test (MWT) were used. Data were analyzed with Statistica 13.1 (Statsoft, Tulsa, OK, USA) software at a significance level of *p* ≤ 0.05.

## 3. Results

### 3.1. Fish Growth

Growth data on experimental fish are presented in Table 3. Differences between initial and final measurements of body length and body weight, FCR, SGR, VSI, and PER were not significantly different for all experimental groups (TT, *p* > 0.05). HSI recorded in S1.0 (3.88%) was higher than those in the experimental group S0.5 (3.74%) and control group (3.46%) in experiment A (TT, *p* ≤ 0.05).

### 3.2. Hematology and Blood Biochemistry Profile

In the RBC parameter, statistical differences were observed only in rainbow trout between the control and experimental groups (E2S0.5—0.84 T L^−1^; E2S1.0—0.84 T L^−1^). In the mean cell volume (MCV) index, significant differences also appeared in rainbow trout between the control group and both experimental groups (E2S0.5—437 fl; E2S1.0—451 fl) and in Siberian sturgeon but only between the control group and the group with higher squalene addition (E1S1.0—143 fl). With the MCH parameter, statistical differences were observed in Siberian sturgeon (E1) and rainbow trout (E2) between the control and both experimental groups (E1S0.5—131 pg; E1S1.0—0.141 pg; E2S0.5 and S1.0—88 pg). No significant differences were found between experimental groups in Hb, PCV, and MCHC in all experiments (TT, *p* > 0.05) (Table 3).

No significant differences were found between the control and experimental groups in all remaining biochemical blood indices for Siberian sturgeon (TT, *p* > 0.05) (Table 3). Significant differences were found between control (3.41 mmol dm^−3^) and experimental groups (2.65–2.46 mmol dm^−3^) observed in TAG level in rainbow trout. CHOL was significantly higher in experimental group S1.0 (6.54 mmol dm^−3^) than in the control group (5.51 mmol dm^−3^) in the Eurasian perch experiment (TT, *p* ≤ 0.05).

### 3.3. Hepatocyte Measurements

**Figure 1:**
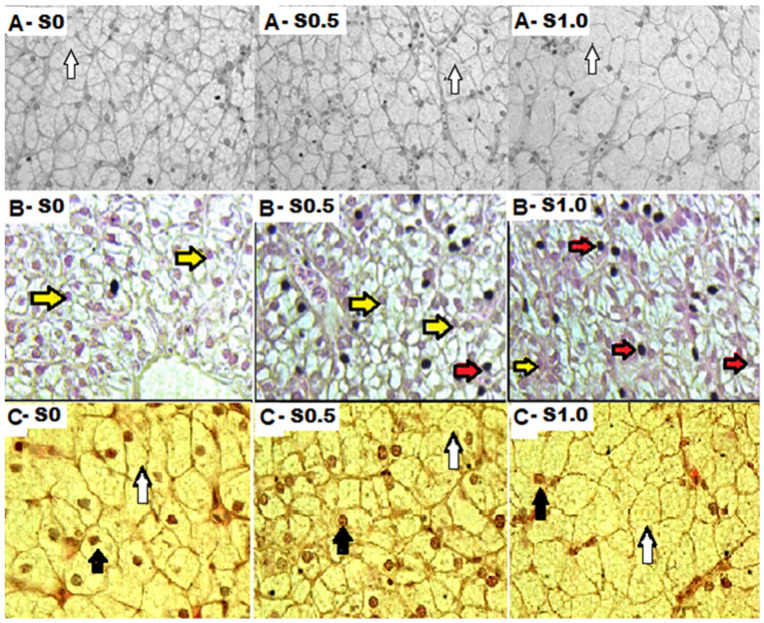
Histological picture of the liver of experimental fish—Siberian sturgeon (A-S0, A-S0.5, A-S1.0), rainbow trout (B-S0, B-S0.5, B-S1.0), Eurasian perch (B-S0, B-S0.5, B-S1.0). Forty (40)× magnification. Yellow or black arrows—normal nuclei, red arrows—pyknotic nuclei, white arrow—fat droplets.

In group S1.0, significantly higher HCD was found when compared to S0 in all experimental fish (TT, *p* ≤ 0.05) (Table 4). This resulted in turn in significantly lower NCPI (TT, *p* < 0.05) for Siberian sturgeon (0.27 µm) and Eurasian perch (0.37 µm). In the group, S1.0 was significantly higher HND (TT, *p* ≤ 0.05) when compared to S0 and S0.5 in the Eurasian perch. A representative image of the liver tissue of the experimental fish is shown in Figure 1. In the obtained image of liver cells, numerous lipid droplets were observed in the hepatocytes of fish fed by squalene feed, mainly in Siberian sturgeon (image A) and in Eurasian perch (image C). A high number of pyknotic nuclei was also noted, mainly in rainbow trout (image B) in the groups supplemented with SQ.

### 3.4. Chemical Analysis and Fatty Acids Profile of Fish Muscle and Liver

Results of the fundamental chemical analysis of the muscle and liver of experimental fish are presented in Table 5. No significant differences between experimental groups in dry matter, crude protein, crude fat, and ash (TT, *p* > 0.05) were found.

**Figure 2:**
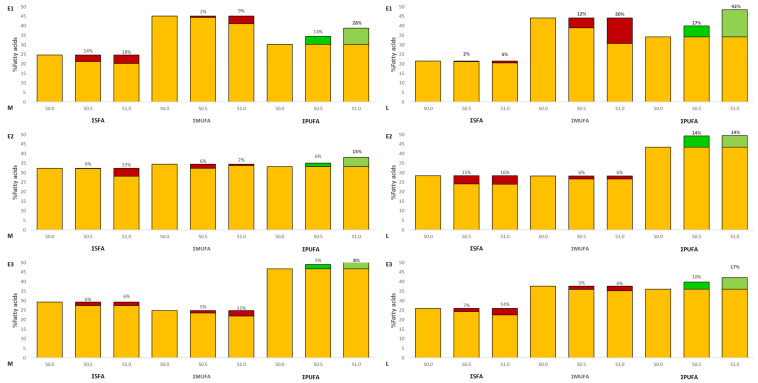
Fatty acids profile content in the fat of the muscle (M) and liver (L) of the experimental fish (Siberian sturgeon E1, rainbow trout E2, Eurasian perch E3). Red indicates a decrease and green indicates an increase in relation to the control group. Bold numbers show columns with significantly different results (*p* ≤ 0.05).

**Figure 3:**
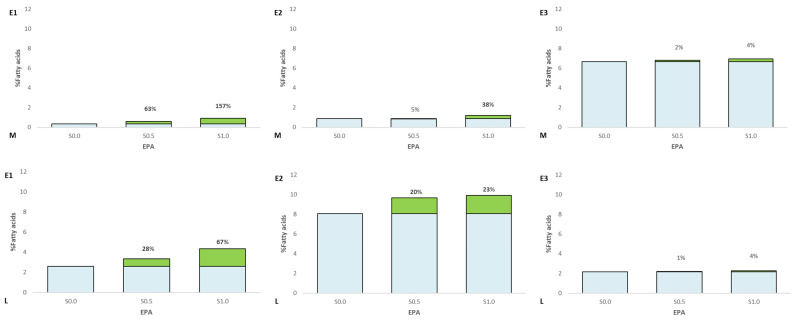
EPA content in the fat of the muscle (M) and liver (L) of the experimental fish (Siberian sturgeon E1, rainbow trout E2, Eurasian perch E3). Green indicates an increase in relation to the control group. Bold numbers show columns with significantly different results (*p* ≤ 0.05).

**Figure 4:**
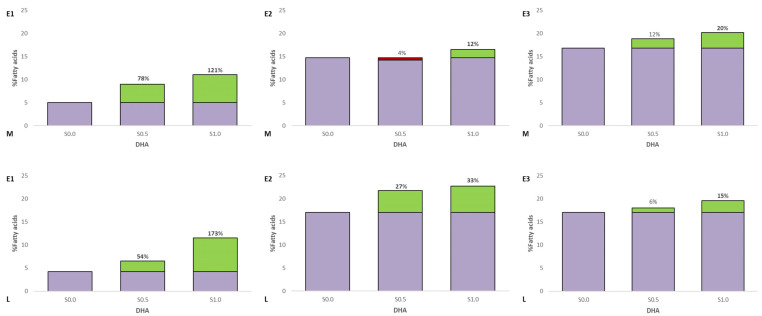
DHA content in the fat of the muscle (M) and liver (L) of the experimental fish (Siberian sturgeon E1, rainbow trout E2, Euroasian perch E3). Red indicates a decrease and green indicates an increase in relation to the control group. Bold numbers show columns with significantly different results (*p* ≤ 0.05).

Fatty acid profiles in the muscle and liver of experimental fish are presented in Figure 2. In experiment 1, growth of PUFA was observed in muscle (S0.5—13.5%, S1.0—27.8%) and liver (S0.5—17.0%, S1.0—42.0%) compared to the control group (TT, *p* ≤ 0.05). A decrease of MUFA was observed in experiment 1 (E1) groups in the liver (S0.5—11.9%, S1.0—30.3%). Statistically significant differences were found in eicosapentaenoic acid (EPA) and docosahexaenoic acid (DHA) in the fat of muscle and liver of the experimental fish (TT, *p* > 0.05) (Figure 3 and Figure 4). See Supplementary Material for details.

In experiment 2, growth of PUFA was observed in muscle in group S1.0 (15.0%) and liver (S0.5—13.5%, S1.0—14.0%) compared to the control group (TT, *p* ≤ 0.05). Statistically significant differences were found in EPA and DHA in the fat of muscle of the experimental fish in group S1.0 (TT, *p* > 0.05). An increase in EPA and DHA was observed in experiment groups in the liver.

In the last experiment, statistically significant differences were found in PUFA and DHA in the fat of muscle and liver in the experimental fish in group S1.0 (TT, *p* > 0.05).

### 3.5. Squalene Retention

Supplementation of squalene at 0.5% and 1.0% levels resulted in its negligible retention in the liver at 0.38% and 0.79% of total oil, respectively. Squalene was detected in Siberian sturgeon muscle (0.21% of total oil) only in the group with the highest (1.16%) squalene content.

## 4. Discussion

In chondrichthyans, squalene accumulates mainly in the liver [35]. The presence of squalene has also been reported in the pancreas, heart, liver, and kidney of sharks [36], as well as in the muscles of some freshwater fish [30].

The hepatosomatic index (HSI) is the most frequently used indicator of liver dysfunction [37]. To assess the metabolic activity of hepatocytes, morphometric parameters such as the number of hepatocytes, surface area, size of nuclei, and number of lipid droplets in the cytoplasm are most commonly used [38]. The addition of 1% of squalene resulted in a statistically significant increase in the hepatosomatic index in fish of the E1S1.0 group (TT, *p* ≤ 0.05) (Table 3) probably due to the increased diameter of hepatocytes (TT, *p* ≤ 0.05) which resulted in decreased NCPI (TT, *p* ≤ 0.05). Research carried out by Bjerkeng et al. [39] showed that the addition of 1% cholesterol in feed increases the size of the HSI index and the amount of cholesterol in the liver of Atlantic salmon. Bigger hepatocytes are seen in the liver of the S1.0 group (Figure 1). The histological picture of the liver of the studied Siberian sturgeons seems to confirm this phenomenon.

The morphological and physiological condition of the fish liver is very often related to nutrition [40,41]. The liver is a good indicator of nutritional pathology because of its function in metabolizing gastrointestinal products. The most common changes observed in the liver are vacuolization of hepatocytes, fat degeneration, changes in the liver parenchyma, and necrosis [28]. The most frequently used indicator of hepatocyte metabolic activity is morphometric parameters: hepatocyte number, surface area, nuclei size, and the content of glycogen and lipids in the cytoplasm [38]. Although the serum levels of liver enzymes in the present study are normal, the histological picture of trout liver is not normal. The hepatocytes of fish fed with fodder supplemented with squalene grew in size (Table 4) and their nuclei more often show pycnotic changes. Pycnosis (karyopycnosis) is the phenomenon of shrinkage or condensation of the cell nucleus with increased density or chromatin packing [42]. Pycnosis most often affects cells undergoing necrosis [43] or apoptosis [44]. Karyopycnosis is often the process that initiates apoptosis or cell death. The hepatocyte nuclei of the tested fish are dark in color (change in metabolic activity), but do not decrease in size, which results from the constant nucleocytoplasmic index in each group. Similar changes in the liver of rainbow trout were observed by Ostaszewska et al. [41] in a study of the effect of casein replacement in feed by soy protein concentrate, where rainbow trout hepatocytes had irregularly shaped pyknotic nuclei and their location in the cell was eccentric. These changes only affected fish fed with fodder with soy protein concentrate.

Red blood cell markers (RBC, MCV, MCH, and MCHC) are important for the assessment of anemia in most animals [45]. In the present study, the statistically significant increase in some of the red blood cell markers in the blood of two exanimated species (E1; E2) was observed (TT, *p* ≤ 0.05). The parameters of RBC, MCH, and MCV showed statistical differences in the blood of both experimental groups of rainbow trout (E2S0,5; E2S01). Moreover, in the blood of Siberian sturgeon, the situation was similar excluding RBC and MCV differences just in the group with a higher level of SQ.

Biochemical parameters can be used to diagnose fish diseases. Triglycerides and cholesterol play very important roles in the body, and their levels depend mainly on the type of food consumed [46]. Triglycerides are the main energy reservoir and their levels in the blood serum are usually used to determine the metabolic state of the body [47]. In the performed experiment, it was observed that the administration of even a dose of squalene as high as 1% did not cause a significant increase in blood cholesterol levels, in the case of rainbow trout (E2) even reduced levels of triglycerides in the blood of that species was observed. Enzymes, including aminotransferases and phosphatases are considered an early warning sign of potentially harmful changes in fish organisms [48]. In the present study, no significant alterations in AST activities were observed (although it tended to increase with SQ level increase) which indicates no severe hepatic dysfunction.

According to Popa et al. [35], the retention of SQ takes place mainly in fish liver. However, in this study no SQ retention was found either in the liver or in the muscles of Eurasian perch fed SQ supplemented feeds. At the same time, a significant increase of blood CHOL by 8.6% (in S0.5) and 15.75% (in S1.0) was noted when compared to the control group (Table 3). No reference data regarding blood CHOL levels in Eurasian perch is available in the literature, but obtained results were two-fold higher than those reported by Zakęś et al. [49] (2.4–2.6 mmol dm^−3^) for pike perch offered feed containing a similar level of crude fat. On the other hand, research by Wang et al. [50], performed on Chinese perch (*Siniperca chuatsi*), has shown that amount of CHOL in the blood serum increases with the increase in fat level in feed. In those studies, 17% crude fat in feed resulted in a much higher CHOL level in the blood serum (7.75 mmol dm^−3^) compared to the results obtained in this study. Studies carried out by Hrubec and Smith [51] showed that the average reference value of the level of CHOL in the serum of yellow perch (*Perca flavescens*) is 13.56 mmol dm^−3^ in the range from 10.11 to 17.94 mmol dm^−3^, and they are twice as high as those obtained in this study (6.03–6.54 mmol dm^−3^). The level of blood CHOL is built by exogenous sources (feed CHOL) and endogenous sources (liver de novo synthesis) [52,53]. In humans, CHOL biosynthesis is regulated in some steps, including the activity of squalene monodoxidase [54], which depends on the squalene level in tissues. In other words, squalene monodoxidase activity is down-regulated by an increase in SQ level. Thus, SQ administered in feeds probably limited SQ de novo synthesis but most of it was converted to CHOL. Therefore, the lack of SQ in Eurasian perch tissues in the conducted study suggests that it has been utilized in the process of CHOL synthesis.

The obtained results of hematological and biochemical indicators and liver histology did not show any harmful effects of squalene on the health status of all experimental fish as well as growth performance.

PUFA are not synthesized de novo in vertebrates due to the lack of delta-12 and delta-15 desaturases, therefore they must be supplied in the diet [55]. Consequently, the level of PUFA in fish muscles depends primarily on the FA profile of the feed [4]. Therefore, it is reasonable to look for new solutions that increase PUFA by protecting them from oxidation.

Dessì et al. [18] indicated that the addition of squalene in a 1:7 ratio (SQ:PUFA) can stabilize the fatty acid profile and protect the PUFA against oxidation by up to 50%, which is associated with the high reactivity of C=C double bonds present in squalene [8]. It can be supposed that the high reactivity of the double bonds found in squalene protected PUFA double bonds against oxidation in vivo.

Our research shows that despite the identical amount of PUFA administered in feed, we recorded a statistically significant increase (TT, *p* ≤ 0.05) in all experimental groups in both the liver and muscles of Siberian sturgeon (Figure 2). In group S0.5, PUFA increased by 13.0% in muscles and 13.3% in the liver, and in group S1.0 by 29.4% and 17.6%, respectively. Experiment 2 (E2) shows that despite the decrease in the amount of PUFA administered in experimental feeds S0.5 and S1.0 (27.68% and 27.32% of the total share, respectively) in comparison to the control (28.39%), we recorded a statistically significant increase (TT, *p* ≤ 0.05) with the increase in the amount of squalene in the studied groups, both in the liver and muscles of rainbow trout (Figure 2). In the S0.5 group, the increase in PUFA by 0.05% in muscles and 0.08% in the liver was noted, and in the S1.0 group by 1.50% and 1.62%, respectively. In experiment 3 (E3), the obtained results showed that despite the lower PUFA content in the experimental feeds S0.5 and S1.0 (16.91% and 16.07% share of the total FA, respectively) compared to the control (17.32%), we recorded a statistically significant increase in PUFA (TT, *p* < 0.05) accompanied by an increase in SQ content in both the liver and muscles of Eurasian perch (Figure 2). Blanchard et al. [5] reported that Eurasian perches (with an average weight of 102 g) fed with PUFA reach feed (20.94–38.82%) exhibited the amount of PUFA in the liver in the range from 25.36 to 43.32%. In the conducted experiment (fish weight 53 g), significantly higher PUFA values in the liver (46.75–50.65%) were obtained, despite the lower amount in feed given (16.07–17.32%) when compared to that reported by Blanchard et al. [5]. Jankowska et al. [31] reported that PUFA in Eurasian perch liver coming from either RAS or wild was ranging between 22.64 and 28.49%, whenever fed with the feed containing 34.18–37.99% of PUFA, also being lower than the one recorded in this study. The obtained results indicate that the increase in the amount of PUFA in the muscles and liver of Eurasian perch is not related to the amount of PUFA administered in the feed.

In the conducted experiment, in addition to the increase in total PUFA, some FAs including DHA were also reported. Maita et al. [56] suggest that in fish, DHA retention is possible mainly in muscles and the liver. This is because the delta-4 double bond is more resistant to oxidative processes [57]. Thus, the DHA content is usually higher than EPA in fish fat. Studies carried out by us show an increase in the amount of DHA and EPA in the muscles and liver of the Siberian sturgeon with the increase in squalene addition (TT, *p* ≤ 0.05) (Figure 3 and Figure 4). In the muscles, in group S0.5, the level of DHA and EPA increased by 78.53% and 61.11%, respectively, and in the S1.0 group 121.07%, and 155.66%, respectively, compared to the control group. In the liver in the S0.5 group, there was an increase of 27.59% and 54.37%, and in the group S1.0 67.05% and 170.45%. DHA in rainbow strand muscles with increasing squalene addition (TT, *p* ≤ 0.05). In the rainbow trout muscles, in group S1.0, the level of DHA and EPA increased by 11.81%, respectively, compared to the control group. In the liver in the S0.5 group, there was an increase of 20.10% (EPA) and 27.44% (DHA), and in the group S1.0 23.08% and 33.29%. In experiment 3, when compared to the control group, the DHA level was higher by 4,15% in muscles in the S1.0 groups, and in the liver in this same group, the DHA content was higher at 19.98% compared to the control. The obtained results indicate that the increase in the amount of DHA in the muscles and liver of Eurasian perch is not related to the amount of DHA administered in the feed but to the amount of supplemented SQ.

The obtained results show the positive effect of squalene on PUFA and DHA levels in muscle and liver in Siberian sturgeon, rainbow trout, and Eurasian perch when it was offered in feed in 0.5 and 1.0% doses. However, if squalene inhibited PUFA oxidation, it is possible that it disturbed the physiological hepatic lipid metabolism which resulted in hepatic lesions, and thus in higher doses it may disturb fish welfare.

## 5. Conclusions

In conclusion, the addition of squalene at doses of 0.5 and 1% to feeds used in the feeding of Siberian sturgeon, rainbow trout, and Eurasian perch did not affect growth indices, although trends were shown for a decrease in feed conversion ratio (FCR) and an increase in relative weight gain (SGR) and protein growth efficiency ratio (PER).

The analysis of the fatty acid profile showed that feed supplementation with squalene increased the amount of n-3 group acids in the liver to a greater extent than in the muscles of the fish studied. Moreover, squalene added to feeds had a greater effect on the content of docosahexaenoic acid (DHA) compared to eicosapentaenoic acid (EPA) in both the muscles and liver of the fish species tested, with a greater effect in the liver than in the muscles.

The study provides the first insight into the involvement of dietary SQ in the lipid metabolism in Siberian sturgeon, rainbow trout, and Eurasian perch suggesting that the addition of SQ had a positive effect on PUFA retention in the tissues. However, because the exact mechanisms standing behind this phenomenon remain at a hypothetical level, and the addition of SQ at higher levels can cause some hepatological lesions, more research is needed aiming at the exploration of the specific pathways involved.

## Figures and Tables

**Table 1:** Ingredients and chemical composition of the control experimental diets.

Formulation, g 100 g^−1^	E1	E2	E3
Fishmeal ^a^	32.00	31.00	20.00
Soybean protein concentrate ^b^	30.00	30.00	30.00
Poultry meal ^c^	0.00	0.00	20.00
Wheat flour ^d^	20.00	20.00	20.00
Cod-liver oil ^e^	0.00	0.00	7.00
Fish oil ^f^	7.50	8.00	0.00
Soybean oil ^g^	7.50	8.00	1.00
Premix ^hij^	3.00	3.00	2.00
Squalene ^k^	0.00	0.00	0.00
Ingredients, g 100 g^−1^			
Dry matter	95.85	95.67	95.50
Crude protein	41.60	41.22	45.90
Crude fat	18.90	20.02	13.40
Crude fiber	1.71	1.63	1.62
NFE	27.72	26.83	26.93
Crude ash	5.92	5.97	7.65
Gross energy (MJ kg^−1^)	17.91	15.52	16.68

^a^ Skaagen, Danemark, ^b^ Hamlet HP 300, Danemark, ^c^ SONAC—Poland, ^d^ SZCZEPANKI—Poland, ^e^ Mollers, Norway, ^f^ Polfish, Poland, ^g^ Artex, Poland, ^h^ Dolfos, Poland, ^k^ SIGMA ALDRICH, Germany. ^i^ Vitamin premix (IU kg^−1^ or mg kg^−1^ dry diet): Vitamin A—70,000 UI kg^−1^; Vitamin D—200,000 UI kg^−1^; Vitamin E—17,500; Vitamin K—867; Vitamin C (ascorbic acid phosphate)—28,500; Vitamin B_1_—1067; Vitamin B_2_—2000; Vitamin B_5_—5334; Vitamin B_6_—1334; Vitamin B_12_—4; Biotin—200, Niacin—12,000, Folic acid—800; Inositol—20,000; Choline chloride—120,000; Betaine—75,000. ^j^ Mineral premix (mg kg^−1^ dry diet): Ferrum (FeSO_4_ ∗ H_2_O)—4334; Iodine (KI)—734; Copper (CuSO_4_ ∗ 5H_2_O)—267; Mangan (MnO)—734; Zinc (ZnSO_4_ ∗ H_2_O)—1250 and Zinc (ZnO)—750; Selen—(Na_2_SeO_4_)—34 mg.

**Table 2:** Fatty acids profile (%) of the control experimental diets (E1–E3). Squalene was calculated as a sum of squalene included in fish oil and fish meal.

Fatty Acids		Feeds	
	E1	E2	E3
C 10:0	0.00	0.00	0.78
C 12:0	0.00	0.00	1.54
C 14:0	9.75	7.96	4.81
C 15:0	0.85	0.73	9.39
C 16:0	19.47	21.70	20.12
C 17:0	0.41	0.38	0.26
C 18:0	3.65	3.95	7.42
C 20:0	0.23	0.31	0.43
C 22:0	0.59	0.49	0.36
ΣSFA	34.96	35.52	45.11
C 14:1	0.45	0.49	0.37
C 16:1	6.84	5.46	4.59
C 17:1	0.57	0.45	2.57
C 18:1 c9	20.32	21.43	12.97
C 18:1 c11	3.09	2.73	4.28
C 20:1 n-9	2.58	2.06	1.61
C 20:1 n-7	0.14	0.10	0.25
C 22:1 n11	1.82	1.43	0.84
C 22:1 n9	0.18	0.13	0.09
ΣMUFA	35.98	35.66	27.58
C 18:2 n-6	11.24	15.23	8.84
C 18:3	2.18	1.84	0.69
C 18:4	0.99	0.70	0.78
C 20:2	0.16	0.11	0.11
C 20:3 n6	0.02	0.01	0.06
C 20:4 n6	0.18	0.13	0.07
C 20:3 n3	0.02	0.01	0.04
C 20:4 n3	0.16	0.11	0.09
C 20:5 n3 (EPA)	1.53	1.12	0.84
C 22:5 n6	0.37	0.28	0.14
C 22:5 n3	0.11	0.08	0.21
C 22:6 n3 (DHA)	12.10	9.20	15.30
ΣPUFA	29.06	28.82	27.32
Squalene	0.16	0.11	0.19
n-3	13.92	10,52	16.48
n-6	11.81	5.65	9.11
n-3/n-6	1.18	1.87	1.81

**Table 3:** Growth performance, hematological indices, blood biochemical parameters and hepatocyte indices of E1–E3 feed of experimental diets for 56 days. Values are presented as mean ± SD. Letter indexes and bold show significantly different results (*p* ≤ 0.05). The presented statistical analysis refers to results within each species.

Indicate	Siberian Sturgeon			Rainbow Trout			Eurasian Perch		
	S0	S0.5	S1.0	S0	S0.5	S1.0	S0	S0.5	S1.0
Initial total length (cm fish^−1^)	27.6 ± 1.7	27.3 ± 1.7	27.8 ± 1.3	27.6 ± 1.7	27.3 ± 1.7	27.8 ± 1.3	11.7 ± 0.7	11.5 ± 0.8	11.6 ± 0.7
Final total length (cm fish^−1^)	40.8 ± 5.0	40.6 ± 3.8	41.0 ± 2.8	40.8 ± 5.0	40.6 ± 3.9	41.0 ± 2.8	14.8 ± 1.0	14.7 ± 0.9	14.9 ± 0.7
Initial weight (g fish^−1^)	68.6 ± 15.5	65.6 ± 12.3	66.7 ± 9.2	68.6 ± 15.5	66.1 ± 12.7	66.7 ± 9.2	24.5 ± 4.4	23.4 ± 4.6	23.4 ± 4.5
Final weight (g fish^−1^)	192.3 ± 47.1	193.4 ± 46.5	209.1 ± 52.7	209.1 ± 53.1	197.2 ± 51.3	193.4 ± 46.5	54.2 ± 5.7	51.9 ± 6.5	52.2 ± 4.1
SGR (%/d)	1.84 ± 0.05	1.87 ± 0.05	1.90 ± 0.08	1.17 ^a^ ± 0.03	**1.24 ^b^ ± 0.04**	**1.24 ^b^ ± 0.04**	1.42 ± 0.09	1.42 ± 0.16	1.43 ± 0.10
FCR	1.09 ± 0.02	1.07 ± 0.04	1.05 ± 0.03	1.09 ± 0.02	1.07 ± 0.04	1.05 ± 0.03	1.62 ± 0.04	1.58 ± 0.27	1.54 ± 0.27
VSI (%)	11.8 ± 2.0	11.8 ± 3.2	11.8 ± 2.5	14.50 ± 2.10	14.50 ± 1.20	14.70 ± 1.70	14.0 ± 1.6	14.8 ± 1.5	15.8 ± 3.0
HSI (%)	3.46 ^a^ ± 0.78	3.74 ^a^ ± 1.04	**3.88 ^b^ ± 0.75**	3.19 ± 1.07	3.74 ± 1.04	3.88 ± 0.75	2.02 ± 0.67	2.09 ± 0.45	2.34 ± 0.75
PER (%)	2.21 ± 0.03	2.24 ± 0.08	2.27 ± 0.07	1.68 ^a^ ± 0.10	1.81 ^a^ ± 0.24	**1.94 ^b^ ± 0.07**	1.37 ± 0.04	1.44 ± 0.24	1.46 ± 0.21
RBC (T L ^−1^)	0.25 ± 0.04	0.23 ± 0.04	0.21 ± 0.06	0.74 ^a^ ± 0.09	**0.84 ^b^ ± 0.10**	**0.84 ^b^ ± 0.01**	0.91 ± 0.16	0.93 ± 0.22	1.10 ± 0.21
PCV (1 L ^−1^)	0.11 ± 0.02	0.11 ± 0.02	0.14 ± 0.07	0.38 ± 0.02	0.36 ± 0.03	0.37 ± 0.03	0.44 ± 0.11	0.42 ± 0.13	0.44 ± 0.09
HB (g L ^−1^)	73.57 ^a^ ± 9.79	74.36 ^a^ ± 8.08	71.78 ^a^ ± 8.32	80.0 ^a^ ± 4.7	73.4 ^a^ ± 11.6	72.4 ^a^ ± 12.03	94.6 ^a^ ± 20.7	105.2 ^a^ ± 19.7	106.6 ^a^ ± 10.6
MCV (fl)	107 ^a^ ± 19	114 ^a^ ± 19	**143 ^b^ ± 69**	521 ^a^ ± 65	**437 ^b^ ± 66**	**451 ^b^ ± 55**	492 ± 120	480 ± 204	407 ± 84
MCH (pg)	119 ^a^ ± 26	**131 ^b^ ± 25**	**141 ^b^ ± 28**	110 ^a^ ± 15	**88 ^b^ ± 14**	**88 ^b^ ± 20**	109 ± 43	118 ± 38	100 ± 24
MCHC (g L^−1^)	0.28 ± 0.06	0.29 ± 0.06	0.29 ± 0.06	0.21 ± 0.01	0.21 ± 0.05	0.20 ± 0.04	0.24 ± 0.07	0.24 ± 0.05	0.25 ± 0.05
GLU (mmol dm^−3^)	3.42 ± 0.46	3.63 ± 0.32	3.77 ± 0.72	6.56 ± 2.23	6.67 ± 1.68	6.56 ± 3.68	5.94 ± 2.01	6.10 ± 2.35	6.57 ± 3.21
TAG (mmol dm^−3^)	12.28 ± 4.28	14.02 ± 4.45	14.04 ± 2.92	3.41 ^a^ ± 1.25	2.65 ^ab^ ± 0.52	**2.46 ^b^ ± 0.77**	8.97 ± 2.53	9.80 ± 3.16	8.01 ± 2.80
CHOL (mmol dm^−3^)	2.79 ± 0.43	3.00 ± 0.61	3.21 ± 0.64	6.51 ± 1.70	6.56 ± 2.20	6.96 ± 1.27	5.51 ^a^ ± 0.56	6.03 ^ab^ ± 0.60	**6.54 ^b^ ± 1.21**
TP (g dm^−3^)	24.00 ± 5.00	25.00 ± 5.00	28.00 ± 6.00	36.9 ± 4.1	36.7 ± 3.9	36.7 ± 5.3	55.8 ± 7.4	51.0 ± 5.5	55.4 ± 8.8
ALB (g dm^−3^)	10.00 ± 2.00	9.00 ± 2.00	10.00 ± 2.00	11.6 ± 1.0	11.3 ± 0.9	12.1 ± 2.7	16.8 ± 1.9	15.8 ± 2.1	17.1 ± 3.1
GLOB (g dm^−3^)	16.00 ± 6.00	15.00 ± 4.00	18.00 ± 5.00	25.3 ± 3.4	25.3 ± 3.3	28.00 ± 3.8	39.0 ± 7.1	35.2 ± 3.8	38.4 ± 8.2
AST (U/L)	210 ± 61	250 ± 114	270 ± 117	237 ± 90	232 ± 110	220 ± 56	127 ± 57	146 ± 87	174 ± 123

**Table 4:** Values of studied liver histological parameters of Siberian sturgeon (E1), rainbow trout (E2), and European perch (E3) fed with squalene-supplemented feeds. Data were presented as mean values ± SD. Letter indexes and bold show significantly different results (*p* ≤ 0.05). The presented statistical analysis refers to results within each species.

Indicate	Siberian Sturgeon			Rainbow Trout			Eurasian Perch		
	S0	S0.5	S1.0	S0	S0.5	S1.0	S0	S0.5	S1.0
**HCD (µm)**	**23.3 ^a^ ± 1.8**	**24.0 ^a^ ± 2.4**	**25.5 ^b^ ± 1.8**	**13.8 ^a^ ± 0.6**	**14.1 ^ab^ ± 0.5**	**14.5 ^b^ ± 0.6**	**14.2 ^a^ ± 0.8**	**14.4 ^a^ ± 0.7**	**15.3 ^b^ ± 1.2**
**HND (µm)**	7.1 ± 0.5	7.2 ± 0.6	6.9 ± 0.3	4.8 ± 0.2	4.9 ± 0.2	4.9 ± 0.2	5.4 ^ab^ ± 0.2	5.3 ^a^ ± 0.2	**5.7 ^b^ ± 0.5**
**NCPI**	0.31 ^a^ ± 0.03	0.30 ^a^ ± 0.04	**0.27 ^b^ ± 0.02**	0.35 ± 0.01	0.35 ± 0.01	0.34 ± 0.01	0.39 ^a^ ± 0.02	0.39 ^a^ ± 0.02	**0.37 ^b^ ± 0.04**

Values in rows marked with other letter subscripts show between-group differences that are statistically significant (*p* ≤ 0.05).

**Table 5:** The final composition of muscle (M) and liver (L) of experimental fish (E1–E3) (n = 9 per group). Results are presented as mean ± SD. Letter indexes show significantly different results (*p* ≤ 0.05) in rows within tissue.

Composition g 100 g^−1^	Muscle			Liver		
	S0	S0.5	S1.0	S0	S0.5	S1.0
Siberian sturgeon						
Dry matter	21.4 ± 0.1	21.3 ± 0.2	21.2 ± 0.2	22.5 ^a^ ± 0.1	22.5 ± 0.1	22.5 ± 0.1
Crude protein	13.6 ± 0.3	13.6 ± 0.3	13.6 ± 0.3	4.2 ± 0.03	4.2 ± 0.02	4.2 ± 0.02
Crude fat	5.6 ± 0.30	5.6 ± 0.4	5.6 ± 0.4	16.2 ± 0.25	16.2 ± 0.25	16.3 ± 0.25
Crude ash	2.2 ± 0.03	2.1 ± 0.02	2.1 ± 0.02	2.2 ± 0.03	2.5 ± 0.03	2.5 ± 0.03
Rainbow trout						
Dry matter	23.1 ± 0.2	23.1 ±0.1	23.1 ± 0.1	21.2 ± 0.5	21.3 ± 0.6	21.5 ± 0.5
Crude protein	17.6 ± 0.4	17.6 ± 0.2	17.6 ± 0.3	6.2 ± 0.3	6.3 ± 0.4	6.3 ± 0.3
Crude oil	4.3 ± 0.4	4.3 ± 0.2	4.3 ± 0.4	13.5 ± 0.5	13.5 ± 0.6	13.7 ± 0.5
Crude ash	1.2 ± 0.1	1.2 ± 0.1	1.2 ± 0.1	1.5 ± 0.3	1.5 ± 0.4	1.5 ± 0.3
Eurasian perch						
Dry matter	22.3 ± 0.1	22.3 ± 0.2	22.0 ± 0.1	22.5 ± 0.1	22.5 ± 0.1	22.5 ± 0.1
Crude protein	18.6 ± 0.3	18.6 ± 0.2	18.5 ± 0.2	4.2 ± 0.03	4.2 ± 0.02	4.2 ± 0.02
Crude oil	2.8 ± 0.3	2.8 ± 0.3	2.8 ± 0.2	16.2 ± 0.3	16.2 ± 0.3	16.3 ± 0.3
Crude ash	0.9 ± 0.0	0.9 ± 0.1	0.9 ± 0.1	2.5 ± 0.03	2.5 ± 0.03	2.5 ± 0.03

## Data Availability

Data is contained within the article or Supplementary Material.

## Supplementary Materials

The following supporting information can be downloaded at: https://www.mdpi.com/article/10.3390/ani13162600/s1, Fatty acids percentage content: Table S1. Fatty acid percentage content in muscle of Siberian sturgeon; Table S2. Fatty acid percentage content in liver of Siberian sturgeon; Table S3. Fatty acid percentage content in muscle of rainbow trout; Table S4. Fatty acid percentage content in liver of rainbow trout; Table S5. Fatty acid percentage content in muscle of Eurasian perch; Table S6. Fatty acid percentage content in liver of Eurasian perch.
